# Impact of carbon nanotubes and graphene on immune cells

**DOI:** 10.1186/1479-5876-12-138

**Published:** 2014-05-21

**Authors:** Marco Orecchioni, Davide Bedognetti, Francesco Sgarrella, Francesco M Marincola, Alberto Bianco, Lucia Gemma Delogu

**Affiliations:** 1 Dipartimento di Chimica e Farmacia, Università degli Studi di Sassari, 07100 Sassari, Italy; 2 Infectious Disease and Immunogenetics Section, Department of Transfusion Medicine, Clinical Center and Trans-National Institutes of Health Center for Human Immunology, National Institutes of Health, Bethesda, MD, USA; 3 Research Branch, Sidra Medical and Research Center, Doha, Qatar; 4 Centre National de la Recherche Scientifique, Institut de Biologie Moléculaire et Cellulaire, Laboratoire d’Immunopathologie et Chimie Thérapeutique, 67000 Strasbourg, France

**Keywords:** Carbon nanotubes, Graphene, Graphene oxide, Nanomedicine, Immune system, Cells, Therapy, Diagnosis

## Abstract

It has been recently proposed that nanomaterials, alone or in concert with their specific biomolecular conjugates, can be used to directly modulate the immune system, therefore offering a new tool for the enhancement of immune-based therapies against infectious disease and cancer. Here, we revised the publications on the impact of functionalized carbon nanotubes (f-CNTs), graphene and carbon nanohorns on immune cells. Whereas f-CNTs are the nanomaterial most widely investigated, we noticed a progressive increase of studies focusing on graphene in the last couple of years. The majority of the works (56%) have been carried out on macrophages, following by lymphocytes (30% of the studies). In the case of lymphocytes, T cells were the most investigated (22%) followed by monocytes and dendritic cells (7%), mixed cell populations (peripheral blood mononuclear cells, 6%), and B and natural killer (NK) cells (1%). Most of the studies focused on toxicity and biocompatibility, while mechanistic insights on the effect of carbon nanotubes on immune cells are generally lacking. Only very recently high-throughput gene-expression analyses have shed new lights on unrecognized effects of carbon nanomaterials on the immune system. These investigations have demonstrated that some f-CNTs can directly elicitate specific inflammatory pathways. The interaction of graphene with the immune system is still at a very early stage of investigation. This comprehensive state of the art on biocompatible f-CNTs and graphene on immune cells provides a useful compass to guide future researches on immunological applications of carbon nanomaterials in medicine.

## Introduction

In the burgeoning area of nanotechnology, scientists are nowadays deeply focusing on the translational application of nanomaterials in medicine. Whereas findings from physics, genetics and immunology have already changed the everyday clinical practice in several fields, nanotechnology is expanding its legacy by implementing approaches aimed at delivering therapeutics [1-3] and developing new diagnostic imaging tools [4-6]. One of the most fascinating frontiers of nanotechnology is the development of nanomaterials for diagnostic and therapeutic purposes at the same time. These materials are indicated as theranostic nanomaterials or nanoparticles [7].

For any translational application of nanotechnology in medicine, a critical step is represented by the assessment of their impact on the immune system, independently of their specific purpose [8]. In fact, following parenteral administration (e.g. intravenous, intramuscular, subcutaneous, etc.), nanomaterials immediately enter in contact with peripheral immune cells either in the blood or in the peripheral tissues.

In this review, we primarily focused on the immunological impact of one of the most studied nanomaterials for biomedicine applications, namely functionalized carbon nanotubes [9,10] (f-CNTs). Non functionalized (pristine) carbon nanotubes are virtually insoluble in water, tend to form intracellular conglomerates and induce considerable cytotoxicities, therefore limiting their application as diagnostic or therapeutic materials. Conversely, their functionalization increases the solubility in biological fluids and enhances nanotube biocompatibility. We considered CNTs as functionalized when they present on their surface any molecule covalently linked or adsorbed. Moreover, we took into consideration graphene, the new promising form of carbon [11]. Graphene is a single-layer two-dimensional sp [2] carbon nanomaterial and it has attracted tremendous attention for its intriguing physical, chemical and mechanical properties [12]. We summarized recent studies on the interaction of pristine graphene and graphene oxide (GO), with immune cells. Carbon nanohorns were also taken into consideration because they share with the above mentioned materials similar chemical characteristics. We considered the heterogeneity among the different functionalizations and described the impact on the major peripheral blood mononuclear cell (PBMC) populations: lymphocytes, monocytes and their differentiated progenies (dendritic cells and macrophages). Furthermore, we discerned lymphocyte subpopulations for each reported studies. Most relevant findings are discussed in the text. The diversity of the nanomaterials here investigated and the large area of immune-related publications make this review a useful compass in the fascinating world of *immune nanotechnology*. With a comprehensive state of the art on functionalized biocompatible carbon nanotubes and graphenes, we aimed at depicting a picture of their interactions with the immune system *per se* and in concert with their specific biomolecule conjugations.

### Studies selection criteria and overview

**Figure 1:**
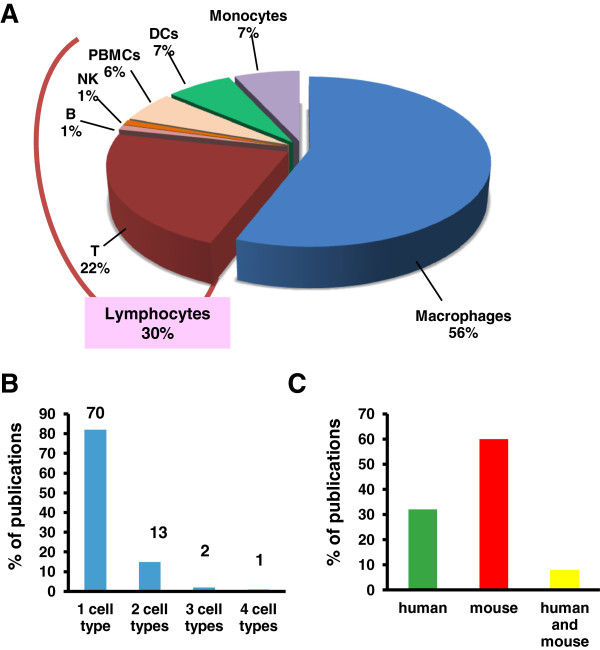
**Overview on cells and animal models of carbon nanomaterials studies. A)** Relative percentages of manuscripts carried out on different immune cell populations. **B)** Percentage of publications according to the number of cell population investigated; each histogram represents the exact number of publications per cell type. **C)** Species examined in the retrieved publications (human, mouse and combination of human and mouse).

**Figure 2:**
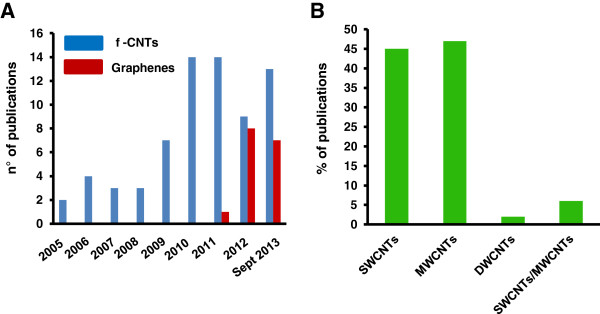
**Status of carbon nanomaterials publications in the last 8 years. A)** Analysis of the amount of publications of f-CNTs and graphenes and their interactions with immune cells (2005 to September 2013). **B)** Percentage of publications based on the different kinds of f-CNTs categorized by their number of graphite layers: single-walled (SWCNTs), double-walled (DWCNTs) and multi-walled (MWCNTs).

To achieve our purpose, we performed a *PubMed* search using the following keywords in different combinations: *functionalized carbon nanotubes, graphene, carbon nanohorns, lymphocytes, T cells, B cells, NK cells, peripheral blood mononuclear cell, dendritic cells, monocytes, macrophages, immune system* and *immune cells.* Keyword exploration was performed one by one and as well as in several different combinations. Study lists reported include all the retrieved publications from 2005 to September 2013. High impact review articles also served as additional tool. To assess what type of cells were most investigated for their interaction with f-CNTs and graphene we summarized the publication according to the cell type (Figure 1A). The majority of the works (56%) have been carried out on macrophages probably because of their extremely important function to attack foreign invader bacteria, viruses and also foreign nanomaterials. Lymphocytes were the second biggest portion of the pie with a 30% of studies. However, we found a huge gap in the different types of lymphocyte populations. T lymphocytes were the most investigated (22%) followed by the mix of cell populations (peripheral blood mononuclear cells, PBMCs), with a 6% of the studies. B cells and NK cells were less investigated with only a 1% of studies each. Studies related to the innate immune cells, others than on macrophages and NK cells, focused on monocytes (7%) and dendritic cells (DCs; 7%). In Figure 1B we show that among 86 publications, the majority of them focused on one cell type (70) and only 1 publication looked at four different immune cell types at the same time. To our knowledge, no investigations have assessed so far on more than four immune cell populations in their interaction with f-CNTs. In Figure 1C we report the number of researches conducted on humans, mice, or both, comprising to *in vitro* or *ex vivo* for human studies and *in vivo* in case of experiments conducted in mouse models. Even though scientists should have caution in translating their findings from mouse to human, our investigation clearly shows that the majority of the studies were conducted on mice (60%), 32% in humans and very few on both (8%). To investigate the degree of scientific interest triggered by different carbon materials, we compared f-CNTs and graphenes (pristine graphene and GO) in terms of number of publications (Figure 2A). In the last years, f-CNTs have been extensively explored for their applications as drug carriers, targeted materials and scaffolds [9]. These works have generated a considerable quantity of ancillary data on their impact on the immune system. Graphene, which has a younger history compared to CNTs, is overall less studied. However, starting from 2012, we noticed an opposite trend, with an increasing number of studies focusing on graphene and GO, resulting in a progressive enrichment of publications dealing with this material in the last couple of years (Figure 2A). This observation reflects the growing interest for graphene among the scientific community. Carbon nanotubes have many structures, differing in length, thickness, and number of graphite layers. In Figure 2B we focused on the different forms f-CNTs categorized by their number of graphite layers: single walled (SWCNTs), double walled (DWCNTs) and multiwalled (MWCNTs). MWCNTs are the most investigated type for their interaction with immune cell (47%), probably because their cost is lower compare to SWCNTs which have been explored in the 45% of studies. A highly useful comparison between the two types of nanotubes was performed in only a small proportion of studies (6%).

### Lymphocytes

Lymphocytes represent about 20-40% of white blood cells (i.e., 70-90% of PBMCs) and are responsible for the antigen-specific (B and T cells) and innate (NK cells) characteristics of immune response. In Additional file 1 we summarized the studies conducted so far evaluating the impact of f-CNTs, graphenes, and carbon nanohorns in these cells [13-39]. One of the first work on CNTs, functionalized by 1,3-dipolar cycloaddition reaction and by oxidation/amidation treatment, was conducted by Dumortier *et al*. in 2006 [16]. The study demonstrated the non toxicity of well functionalized CNTs on primary mouse T and B cells. A year later, the group of Dai, while studying a specific siRNA delivery system against CXCR4 (a critical receptor used by HIV to infect target cells), revealed the non toxicity of pegylated CNTs on human T cell lines and primary T cells [17]. Delogu *et al.*[21] focusing on the use of CNTs for drug delivery to knock-down PTPN22, a gene related to type 1 diabetes [40], confirmed the non toxicity of PEGylated CNTs on T cells. Similar results were also found by Cato *et al.*[18]. A systematic genotoxic study [23] reported the effect of SWCNTs, MWCNTs, and amidated SWCNTs on cultured human lymphocytes. This investigation showed that, in contrast to non functionalized CNTs, the amidation of SWCNTs did not disturb the T cell proliferation, therefore preserving T cells from the observed genotoxicity. The studies discussed above highlights that different type of functionalizations such as 1,3-dipolar cycloaddition, adsorption with phospholipids and polyethylene glycol, and amidation are fundamental for preserving viability of treated cells. Furthermore, other than addressing biocompatibility issues, Fadel *et al.* suggested that f-CNTs can be potentially used in the context of immunotherapy [19]. They proved that anti-CD3 adsorbed onto SWNTs bundles stimulate T cell lines more effectively than equivalent concentrations of soluble anti-CD3. However, the stimuli was more efficient with functionalized versus non functionalized nanotubes [25]. The groups of Bianco and Gennaro demonstrated that two different conjugates f-CNTs and amphotericin B can achieve an antifungal activity comparable to, or better than, amphotericin alone in T cell lines (jurkat cells) [27]. A large number of studies took advantage of the easiness of culturing cell lines such as jurkat cells [13-15,18,21,26-28,39], a line established from the peripheral blood of a 14 year old child affected by T cell leukemia. The clear advantages in using this cell model (highly reproducible experiments, low cost and favorable culture conditions) are somehow in contrast with its neoplastic characteristics that may not always reflect the *in vivo* physiological condition of T cell behavior. To avoid this concern, in a recent study, we explored the impact of different functionalized MWCNTs on PBMCs from healthy donors [31]. Two types of multi-walled carbon nanotubes (of low, 9.5 nm, and big, 20–30 nm, diameter) were initially oxidized and further modified by 1,3-dipolar cycloaddition reaction to obtain ammonium-functionalized nanotubes. After having excluded *in vitro* toxicity and confirmed their dose-depending uptake, we observed that f-CNTs with smaller diameter were internalized more efficiently. Moreover, by analyzing CD4+ and CD8+ T cells, B cells, NK cells, and monocytes, we surprisingly noticed that f-CNTs have a cell specific effect. In fact, none of them induced T cell expansion or activation (evaluated by CD25, and CD69 markers), while all of them were able to activate NK cells, which upregulated CD69 and CD161 activation markers following f-CNTs treatment. Our study [31], which was the first one exploring the impact of nanotubes on natural killer cells, suggests that the use of these f-CNTs could be explored as tool for NK expansion, for example in pre-clinical model of NK-based adoptive therapy. Similarly, the up-regulation of CD25 and the production of IL-6 by CD14+ monocytes upon treatment with ammonium-functionalized CNTsrevealed the activatory properties of these nanotubes (see discussion in the following section) on innate immune cells. Few research groups have started to look at the impact of graphene and GO on the immune system. The group of Liu showed that the complex between GO and anti-IL10 receptor antibodies elicited LPS-stimulated CD8 T cell responses, which makes this functionalized graphene suitable for reprogramming suppressive tumor environment in experimental models [34]. The first extensive investigation on pristine and GO to assess hemocompatibility was performed by Sasidharan *et al.*[35]. The authors observed a significant release of IL-8 and IL-6 in pristine graphene treated PBMC, while GO was able to determine only a moderate release of IL-8. No modulation of the other inflammatory cytokines (IL1B, IL1, TNF, and IL12) was observed. Importantly, the authors showed an excellent compatibility of both types of graphene in multiple assays assessing hemolysis, platelet aggregation and coagulation. The group of Cui addressed whether the coating of GO with polyvinylpyrrolidone (PVP) could increase biocompatibility GO with human immune cells, including T lymphocytes [38]. Besides confirming the GO dose-dependent toxicity on T cells, the authors showed that the induction of apoptosis in these cells was much less pronounced when PVP-coated GO was used [38].

### Monocytes

Monocytes in the blood of healthy people are the 10-30% of PBMCs. Monocytes and their differentiated progeny (macrophages and dendritic cells) play an important role in both the innate and adaptive immunity, by exerting immune-regulatory proprieties to the production of several modulatory cytokines. These cells phagocyte material for two purposes: i) to eliminate waste, and ii) to destroy invading pathogens [41]. During a potential injection of nanomaterials for diagnostic or therapeutic purposes, monocytes will be the responsible population for a possible innate response of the immune system. It should be mentioned that different nanomaterial coating and functionalization are finalized not only to reach a good biocompatibility but also to elude phagocytic-mediated clearance. In this context a good example is given by the work performed by the group of Tasciotti [42], in which the authors showed how nanoporous silicon particles coated with cellular membranes can avoid opsonization, delay uptake by mononuclear phagocytes and elude lysosomal pathway. However, the elicitation of monocyte functions is desirable when the purpose is to enhance the immunogenicity of a certain molecule. In Additional file 2 we report the publications related to the effects of the different carbon materials on monocytes [31,39,43-47].

One of the first works carried out on monocytes was conducted by Meunier *et al.*[43]. By assessing the effect of pristine double-walled CNTs on monocytes, the authors observed an induced IL-1beta secretion linked to caspase-1 and to Nlrp3 inflammasome activation in human monocytes, while no induction of the corresponding mRNA was observed. The authors also showed that similar increase of IL-1β was observed using oxidized DWCNTs. However, the researchers did not assess whether the IL-1beta release mediated by oxidized CNTs was dissociated from the induction of caspase-mediated apoptotic stimuli. In absence of functionalization, in fact, CNTs are highly cytotoxic and can mediate the induction of several inflammatory cytokines through the activation of pathways associated to oxidative stress and caspase cascade [48]. We found, however, that MWCNTs oxidized and functionalized with ammonium group by 1,3-dipolar cycloaddition induce the expression of the CD25 activation markers and release of IL1beta, IL6, TNF, and IL10 by human monocytes not accompanied by the activation of cytotoxic mechanisms [31,39]. Recently, we provided more molecular insight into down-stream events induced by f-CNTs [39]. Through a genome-wide analysis on THP1 monocyte cell lines, we showed that ammonium-functionalized as well as oxidized MWCNTs with larger diameter provoke a profound modulation of immune-regulatory pathways not accompanied by the induction of apoptotic pathways. We demonstrated that these f-CNTs induce profound modulation of inflammatory molecules at the transcriptomic level. Molecular pathways activated by these nanotubes include toll-like receptor (TLR), IL-6, dendritic cell maturation, TNF, NFKB, and T helper 1 chemokine pathways (CXCR3 and CCR5 ligand pathways) [39]. Because of the critical role of these inflammatory pathways (especially the T helper 1 chemokine pathways) in controlling immune-mediated tumor rejection, our findings suggest the highly promising application of this type of f-CNTs as adjuvant molecules in the contest of cancer immunotherapy [49-52]. In contrast, thinner oxidized MWCNTs down-modulated genes associated with ribosomal proteins in both monocyte and T cell lines. Gul-Uludag *et al.*[46] analyzed THP1 cells using fluorescein isothiocyanate (FITC)-labeled magnetic carbon nanotubes. Interestingly, the authors noticed an extremely high uptake of this f-CNTs into this type of cells after 1 hour, whereas localization into nucleus was observed 6 hours after their administration. A recent deep evaluation of a wide variety of f-CNTs showed that compared to raw MWCNTs, anionic functionalization (carboxylated and PEGylated) decreased the production of cytokines/growth factors by THP1 cells (IL-1beta) or by THP1-cocultured with bronchial epithelial cell lines (PDGF-AA, TGF-beta), while neutral and low degree of CNT cationic functionalization showed intermediary effects [47]. In particular, carboxylated MWCNTs did not induce any inflammatory responses. However, high degree of CNT cationic functionalization (PEI) induced the greatest cytokine response, which was also associated to the development of pulmonary fibrosis following nasopharingeal installations in mouse models. No significant fibrosis development was observed in animals treated with anionic nanotubes. Moreover, Wang *et al.*[45] found that well dispersed MWCNTs by serum bovine albumin elicited a more robust IL-1beta production on THP1. Regarding graphene, studies have been focused on macrophages and dendritic cells rather than on monocytes (see following paragraphs).

### Macrophages

Macrophages represent the differentiated counterpart of monocytes in the peripheral tissue. For their phagocytic characteristic and their pilot role in innate immunity, interaction between macrophages and carbon nanotubes has aroused great interest. A 56% of the studies here analyzed were carried out on this immune cell population. A possible reason of the large amount os studies is that this cell type is an easy long-term culturing primary immune cell population compare to T cells or natural killer cells. Additional file 3 gives an overview on the papers published in the last years on macrophage and their interaction with carbon nanotubes, carbon nanohorns, graphene and GO [16,22,29,35,38,45,47,53-103]. The large majority of the work performed *in vitro* was carried out using the murine macrophage cell line RAW 264.7, which can guarantee high experimental reproducibility, it is easy to culture and, being a good transfection host, it is suitable for nanomaterial-based conjugate uptake assays. Dutta *et al.* investigated the importance of plasma protein adsorption by oxidized SWCNTs [54] using RAW 264.7. Intriguingly, the authors noticed that the complex between SWCNT and albumin inhibited the induction of cox-2 by LPS and suggested that the proteins adsorbed onto nanotubes may alter their immune modulatory and toxicity properties. Furthermore, other modifications (e.g. non anionic surfactant coating) could reduce albumin adsorption and decreased their anti-inflammatory properties. The work of Zhou *et al.*[63] elucidated mechanisms of intracellular compartmentalization of functionalized SWCNTs by corroborating and expanding previous observations [55,104]. Pantarotto *et al.*[104] had previously shown that f-CNTs are able to cross the cell membrane and to accumulate into the cytoplasm or reach the nucleus of living cell, while Porter *et al.*[55] observed that SWCNT can cross the membrane of macrophage, localize into the lysosomes and enter the nucleus. In their work, Zhou *et al.*[63] studied in detail the cellular localization of SWNT surface-modified with phospholipids and polyethylene glycol (PL-PEG). The authors reported that these functionalized SWCNTs localize inside mitochondria if they cross the cell membrane or inside lysosomes when they are endocytosized. A more recent study also found similar results for carbon nanohorns that are preferentially localized into macrophage lysosomes opening future applications relying on lysosomes as target organelles for drug delivery or imaging [79]. Dong *et al.*[77] tested pristine SWCNTs and acid-functionalized SWCNTs showed on RAW 264.7 that these materials could damage mitochondrial function and proteasome.

Regarding carbon nanohorns, Lacotte *et al.*[103] found that exposed macrophage increased the production of reactive oxygen species and pro-inflammatory cytokines. The authors suggested that a moderate level of inflammation could be exploited as an intrinsic carbon nanohorn adjuvant function for biomedical applications requiring some activation of the immune system. In the context of graphene, the number of publications related to the interaction of this material and the immune system are increasing. It has been demonstrated that pristine graphene can induce cytotoxicity on macrophage through the depletion of the mitochondrial membrane potential and the increase of intracellular reactive oxygen species [85]. Chen *et al.*[86] described instead the relations with GO demonstrating that it is able to simultaneously trigger autophagy and toll like receptors 4/9-regulated inflammatory responses. Moreover, the authors reported that autophagy was at least partly regulated by the toll-like receptor (TLRs) pathway. The work of Sasidharan *et al.*[35] gave a full picture of bio- and hemocompatibility of both pristine graphene and GO on RAW 264.7 cells and human primary blood components. All the results suggested that the toxicity of pristine graphene can be easily averted by surface modification (i.e. GO). Recently, by focusing on the effect of GO sheet lateral dimensions on human primary monocyte-derived macrophages and murine intraperitoneal macrophages, we demonstrated that the more the lateral dimensions of GO were reduced, the higher were the uptake and the impact of functionality [101]. Li *et al.*[99] tried to understand the interaction between graphene and cell membranes of primary human keratinocytes, human lung epithelial cells and murine macrophages. The authors showed that the entry is initiated at the asperities that are abundant along the materials making clear that local piercing initiates membrane propagation along the extended graphene edge. Overall, there are very few data present for graphene and GO keeping the door open to more mechanistic studies *in vitro, ex vivo* and *in vivo.*

### Dendritic cells

Dendritic cells are specialized antigen-presenting cells that circulate in the blood – where they represent 1-2% of PBMCs- but are preferentially found in lymphoid and peripheral organs. As showed in Figure 1A, studies performed on dendritic cells are considerable less in number as compared to those performed on other antigen-presenting cells. In Additional file 4 we summarized the publications where the authors report findings on DCs and their interaction with different materials [32,33,36,38,53,59,102]. Tkatch *et al.*[32] investigated *in vivo* and *in vitro* the effect of SWCNTs produced by the high-pressure CO disproportionation (HipCO nanotubes). The authors showed that this type of SWCNTs can induce pulmonary inflammation. The investigators observed that SWCNTs also facilitated the recruitment of DCs in lung tissues, while they were unable to interfere with DC activation (assessed through the measurement of CD80, CD86, CD40 and MHC class II molecules). Surprisingly, SWCNT-treated DCs suppressed T cell proliferation response. Different types of functionalization can dramatically change the recognition profile by DCs with consequent different effect on processing and antigen presentation on T cells [32]. Accordingly, the same group [59] demonstrated that coating SWCNTs with anionic phospholipids (phosphatidylserine and diacylphophatidylglycerol) can dramatically enhance their recognition and uptake by phagocytes, such DCs. The authors suggested that a phosphatidylserine coating can be utilized for targeted delivery with specified cargoes into professional phagocytes. Villa *et al.*[33] proved this hypothesis coating CNTs with Wilm’s tumor protein 427 peptide (a vaccine tested in several clinical trials). Immunization of BALB/c mice with the SWCNT-peptide constructs, mixed with immunological adjuvants, induced specific IgG responses against the peptide, proving that CNTs are able to serve as antigen carriers for delivery into antigen presenting cells (APCs).

Data on the effect of graphene on DCs are negligible. Tkach *et al.*[36] showed that GO impairs the stimulatory potential of DCs by decreasing the intracellular levels of LMP7 immunoproteasome subunits required for processing of protein antigen. Polyvinylpyrrolidone (PVP) coating on GO was recently investigated [38]. The functionalization exhibited lower immunogenicity compared with pure GO in inducing differentiation and maturation. In the same work, other experiments performed on T cells and macrophages suggest that PVP-coating have a good immunological biocompatibility and immune enhancement effects *ex vivo.* However, no papers are present in the literature carrying out a deep toxicology analysis on this population. Moreover, carbon nanotube interaction has been investigated mostly in their single-walled form and no data are available for carbon nanohorns.

## Conclusions

Carbon nanotubes have been investigated with great expectation by several research groups in many contexts, ranging from electronics to medicine. Through the years, however, their cytotoxic effects raised major concerns regarding the real possibility to apply carbon nanotubes in medicine as diagnostic or therapeutic applications. Pristine nanotubes in fact tend to form agglomerate, induce cellular necrosis/apoptosis and oxidative stress when ingested by phagocytes. In mice, they determine formation of granulomas, resembling in part the effect of asbestos [48,105]. Moreover, carbon nanotubes can directly bind some plasma protein as fibrinogen and apolipoproteins and trigger activation of complement cascade [106]. Concerning the cytotoxicity, in general, higher is the dose higher is the toxic effect. The functionalization of pristine nanotubes is clearly the key for improving their biocompatibility [107]. As described above, several studies have demonstrated that some f-CNTs bear immunostimulatory properties in absence of cytotoxicity. Importantly, most of the studies have shown lack of relevant toxicity of f-CNTs at relatively high concentrations both *in vitro* and *in vivo*. However, several parameters as the number of walls, the diameter, the length, and, importantly, the type of functionalization can influence their cellular uptake and/or toxicity. Moreover, the route of administration is also a critical factor. For example, lung toxicity has been observed for both pristine and functionalized carbon nanotubes [108]. Toxicity of carbon nanotubes and regulatory aspects has been thoroughly revised elsewhere [109].

Graphene and GO seem to retrace the same road of scientific expectation of CNTs. Even for this material a careful assessment of toxicity *in vitro* and *in vivo* in appropriate models is necessary before starting testing in clinical trials [110]. Risk to benefit ratio needs to be accurately evaluated before any medical application i.e. as delivery tool, immunotheraphy and for biomarker detection [111-113].

Even though there are many technical advantages in using cell lines as model, more studies assessing perturbation in primary human cells are needed. Studies performed on healthy donors are carried out in most of the case separating red blood cells from PBMCs. PBMCs contain monocytes, T cells, B cells, NKs, and dendritic cells. A more profound evaluation of the interaction of some types of population is warranted. NKs and B cells remain very poorly investigated. Studies directed at elucidating the effects of carbon nanomaterials on specific lymphocyte or monocyte subpopulations are lacking. For example, it is totally missed the evaluation of monocyte subpopulation, as classical monocytes CD14+ CD16-, and non-classical monocytes with low level expression of CD14 and high co-expression of the CD16.For T cell studies, taking in consideration T regulatory cells and different stage of T cell maturations are desired.

Moreover, we noticed an almost absence of comparative data on many immune cell types at the same time. Looking at different cell type simultaneously would provide more insight into putative cell-type restricted effects of certain nanomaterials.

It is well known for carbon nanotubes that length and functionalization can lead to very difference reactions on cells, and it is of critical importance to differentiate carbon nanotubes in terms of physical and chemical characteristics. Learning from CNT literature, scientists should be aware to apply this state of mind also to the new born graphene, where lateral dimension and functionalization most likely can make huge difference in immune cells behaviors.

Here we showed that a considerable number of publications combining *in vitro* and *in vivo* data were carried out in mice models. Results of pre-clinical studies in mouse, however, should be taken with caution considering their profound difference in physiological immune responses compare to humans [114]. Regarding gene expression data, we found very few works employing a high throughput design. Whole genome gene-expression is able to give an overall picture of the molecular changes following experimental perturbation. This approach was carried out by Chou *et al.*[48] to investigate the effect of non-functionalized SWCNTs on THP1 derived macrophages using one type of nonfunctionalized nanotubes. Using the same powerful tool we recently provided a precise portrait of the molecular perturbation induced by f-CNTs on T and monocytes human cell lines [39]. As described above, we found that the main effect of MWCNTs (oxidized and further functionalized with ammonium groups) is the induction of specific inflammatory molecular pathways on monocytes, while no relevant immunologic effect on T cells were observed. However, functionalized MWCNTs of low diameter but lacking ammonium group were unable to induce immune activation. Rather, they determine profound modulation on genes encoding ribosomal proteins in both monocytes and T cells. Altogether, these results suggest that some f-CNTs bear an intrinsic immune-modulatory propriety and that this effect is cell specific. These immune-modulatory f-CNTs emerge as potential adjuvant for immune-based therapies against infectious disease and cancer [50]. Notably, molecular pathways activated in monocytes after f-CNT stimulation resemble somehow those that can be obtained through the activation of TLRs, which recognize pathogen-associated molecular patterns. Importantly, mononuclear phagocytes express much higher level of TLRs than other cells and could in part explain the inability of those f-CNTs to induce the same profound immune-modulation on T cells. Although activation of TLRs is plausible, this mechanism has not been conclusively demonstrated yet. However, several TLRs exist, and mechanistic studies aimed at defining the molecular target of f-CNTs represent an urgent subject. In conclusion, many observations suggest that CNTs and nanohorns can exert an immune modulatory effect, but their physical molecular interaction need to be elucidated. On the other hand, the very limited amount of works available in the literature for graphene make this area of research one of the more enthusiastic challenge in the near future nanomedicine scenario.

## Competing interests

The authors declare that they have no competing interests.

## Authors’ contributions

MO carried out the bibliography search. MO, DB, FS, FMM, AB and LGD analyzed the publication data. DB, AB, and LGD drafted the manuscript. FS and FM critically revised the manuscript and figures. All authors read and approved the final manuscript.

## Supplementary Material

Additional file 1 Functionalized carbon nanotubes and graphene on Lymphocytes. Click here for file

Additional file 2 Functionalized carbon nanotubes and graphene on Monocytes. Click here for file

Additional file 3 Functionalized carbon nanotubes and graphene on Macrophages. Click here for file

Additional file 4 Functionalized carbon nanotubes and graphene on Dendritic cells (DCs). Click here for file

## References

[B1] Peer D Karp JM Hong S Farokhzad OC Margalit R Langer R Nanocarriers as an emerging platform for cancer therapy Nat Nanotechnol 2007 2 751 760 10.1038/nnano.2007.387 18654426

[B2] Buxton DB Nanomedicine for the management of lung and blood diseases Nanomedicine (Lond) 2009 4 331 339 10.2217/nnm.09.8 19331540 PMC6568266

[B3] Jain KK Role of nanotechnology in developing new therapies for diseases of the nervous system Nanomedicine (Lond) 2006 1 9 12 10.2217/17435889.1.1.9 17716203

[B4] Delogu LG Vidili G Venturelli E Ménard-Moyon C Zoroddu MA Pilo G Nicolussi P Ligios C Bedognetti D Sgarrella F Manetti R Bianco A Functionalized multiwalled carbon nanotubes as ultrasound contrast agents Proc Natl Acad Sci U S A 2012 109 16612 16617 10.1073/pnas.1208312109 23012426 PMC3478634

[B5] Hellebust A Richards-Kortum R Advances in molecular imaging: targeted optical contrast agents for cancer diagnostics Nanomedicine (Lond) 2012 7 429 445 10.2217/nnm.12.12 22385200 PMC3327054

[B6] Shilo M Reuveni T Motiei M Popovtzer R Nanoparticles as computed tomography contrast agents: current status and future perspectives Nanomedicine (Lond) 2012 7 257 269 10.2217/nnm.11.190 22339135

[B7] Wang LS Chuang MC Ho JA Nanotheranostics–a review of recent publications Int J Nanomedicine 2012 7 4679 4695 22956869 10.2147/IJN.S33065 PMC3431969

[B8] Dobrovolskaia MA Germolec DR Weaver JL Evaluation of nanoparticle immunotoxicity Nat Nanotechnol 2009 4 411 414 10.1038/nnano.2009.175 19581891

[B9] Bianco A Kostarelos K Prato M Making carbon nanotubes biocompatible and biodegradable Chem Commun (Camb) 2011 47 10182 10188 10.1039/c1cc13011k 21776531

[B10] Kostarelos K Bianco A Prato M Promises, facts and challenges for carbon nanotubes in imaging and therapeutics Nat Nanotechnol 2009 4 627 633 10.1038/nnano.2009.241 19809452

[B11] Bussy C Ali-Boucetta H Kostarelos K Safety Considerations for Graphene: Lessons Learnt from Carbon Nanotubes Acc Chem Res 2012 46 692 701 23163827 10.1021/ar300199e

[B12] Geim AK Novoselov KS The rise of graphene Nat Mater 2007 6 183 191 10.1038/nmat1849 17330084

[B13] Wu W Wieckowski S Pastorin G Benincasa M Klumpp C Briand JP Gennaro R Prato M Bianco A Targeted delivery of amphotericin B to cells by using functionalized carbon nanotubes Angew Chem Int Ed Engl 2005 44 6358 6362 10.1002/anie.200501613 16138384

[B14] Bottini M Bruckner S Nika K Bottini N Bellucci S Magrini A Bergamaschi A Mustelin T Multi-walled carbon nanotubes induce T lymphocyte apoptosis Toxicol Lett 2006 160 121 126 10.1016/j.toxlet.2005.06.020 16125885

[B15] Bottini M Cerignoli F Dawson MI Magrini A Rosato N Mustelin T Full-length single-walled carbon nanotubes decorated with streptavidin-conjugated quantum dots as multivalent intracellular fluorescent nanoprobes Biomacromolecules 2006 7 2259 2263 10.1021/bm0602031 16903668

[B16] Dumortier H Lacotte S Pastorin G Marega R Wu W Bonifazi D Briand JP Prato M Muller S Bianco A Functionalized carbon nanotubes are non-cytotoxic and preserve the functionality of primary immune cells Nano Lett 2006 6 1522 1528 10.1021/nl061160x 16834443

[B17] Liu Z Winters M Holodniy M Dai H siRNA delivery into human T cells and primary cells with carbon-nanotube transporters Angew Chem Int Ed Engl 2007 46 2023 2027 10.1002/anie.200604295 17290476

[B18] Cato MH D'Annibale F Mills DM Cerignoli F Dawson MI Bergamaschi E Bottini N Magrini A Bergamaschi A Rosato N Rickert RC Mustelin T Bottini M Cell-type specific and cytoplasmic targeting of PEGylated carbon nanotube-based nanoassemblies J Nanosci Nanotechnol 2008 8 2259 2269 10.1166/jnn.2008.501 18572636 PMC12974605

[B19] Fadel TR Steenblock ER Stern E Li N Wang X Haller GL Pfefferle LD Fahmy TM Enhanced cellular activation with single walled carbon nanotube bundles presenting antibody stimuli Nano Lett 2008 8 2070 2076 10.1021/nl080332i 18547120

[B20] Ochoa-Olmos OE Montero-Montoya R Serrano-Garcia L Basiuk EV Genotoxic properties of nylon-6/MWNTs nanohybrid J Nanosci Nanotechnol 2009 9 4727 4734 10.1166/jnn.2009.1097 19928141

[B21] Delogu LG Magrini A Bergamaschi A Rosato N Dawson MI Bottini N Bottini M Conjugation of antisense oligonucleotides to PEGylated carbon nanotubes enables efficient knockdown of PTPN22 in T lymphocytes Bioconjug Chem 2009 20 427 431 10.1021/bc800540j 19243140

[B22] Zeinali M Jammalan M Ardestani SK Mosaveri N Immunological and cytotoxicological characterization of tuberculin purified protein derivative (PPD) conjugated to single-walled carbon nanotubes Immunol Lett 2009 126 48 53 10.1016/j.imlet.2009.07.012 19664657

[B23] Cveticanin J Joksic G Leskovac A Petrovic S Sobot AV Neskovic O Nanotechnology 2010 21 015102 10.1088/0957-4484/21/1/015102 19946169

[B24] Hu X Cook S Wang P Hwang HM Liu X Williams QL In vitro evaluation of cytotoxicity of engineered carbon nanotubes in selected human cell lines Sci Total Environ 2010 408 1812 1817 10.1016/j.scitotenv.2010.01.035 20167353

[B25] Fadel TR Look M Staffier PA Haller GL Pfefferle LD Fahmy TM Clustering of stimuli on single-walled carbon nanotube bundles enhances cellular activation Langmuir 2010 26 5645 5654 10.1021/la902068z 19764784

[B26] Sabuncu AC Kalluri BS Qian S Stacey MW Beskok A Dispersion state and toxicity of mwCNTs in cell culture medium with different T80 concentrations Colloids Surf B Biointerfaces 2010 78 36 43 10.1016/j.colsurfb.2010.02.005 20236807

[B27] Benincasa M Pacor S Wu W Prato M Bianco A Gennaro R Antifungal activity of amphotericin B conjugated to carbon nanotubes ACS Nano 2011 5 199 208 10.1021/nn1023522 21141979

[B28] Delogu LG Stanford SM Santelli E Magrini A Bergamaschi A Motamedchaboki K Rosato N Mustelin T Bottini N Bottini M Carbon nanotube-based nanocarriers: the importance of keeping it clean J Nanosci Nanotechnol 2010 10 5293 5301 10.1166/jnn.2010.3083 21125885

[B29] Kim JE Lim HT Minai-Tehrani A Kwon JT Shin JY Woo CG Choi M Baek J Jeong DH Ha YC Chae CH Song KS Ahn KH Lee JH Sung HJ Yu IJ Beck GR Jr Cho MH Toxicity and clearance of intratracheally administered multiwalled carbon nanotubes from murine lung J Toxicol Environ Health A 2010 73 1530 1543 10.1080/15287394.2010.511578 20954079

[B30] Sun Z Liu Z Meng J Meng J Duan J Xie S Lu X Zhu Z Wang C Chen S Xu H Yang XD Carbon nanotubes enhance cytotoxicity mediated by human lymphocytes in vitro PLoS One 2011 6 e21073 10.1371/journal.pone.0021073 21731651 PMC3120825

[B31] Delogu LG Venturelli E Manetti R Pinna GA Carru C Madeddu R Murgia L Sgarrella F Dumortier H Bianco A Ex vivo impact of functionalized carbon nanotubes on human immune cells Nanomedicine (Lond) 2012 7 231 243 10.2217/nnm.11.101 22106855

[B32] Tkach AV Shurin GV Shurin MR Kisin ER Murray AR Young SH Star A Fadeel B Kagan VE Shvedova AA Direct effects of carbon nanotubes on dendritic cells induce immune suppression upon pulmonary exposure ACS Nano 2011 5 5755 5762 10.1021/nn2014479 21657201 PMC3170729

[B33] Villa CH Dao T Ahearn I Fehrenbacher N Casey E Rey DA Korontsvit T Zakhaleva V Batt CA Philips MR Scheinberg DA Single-walled carbon nanotubes deliver peptide antigen into dendritic cells and enhance IgG responses to tumor-associated antigens 5311 2011 5 5300 10.1021/nn200182x PMC3143710 21682329

[B34] Ni G Wang Y Wu X Wang X Chen S Liu X Graphene oxide absorbed anti-IL10R antibodies enhance LPS induced immune responses in vitro and in vivo Immunol Lett 2012 148 126 132 10.1016/j.imlet.2012.10.001 23064239

[B35] Sasidharan A Panchakarla LS Sadanandan AR Ashokan A Chandran P Girish CM Menon D Nair SV Rao CN Koyakutty M Hemocompatibility and macrophage response of pristine and functionalized graphene Small 2012 8 1251 1263 10.1002/smll.201102393 22334378

[B36] Tkach AV Yanamala N Stanley S Shurin MR Shurin GV Kisin ER Murray AR Pareso S Khaliullin T Kotchey GP Castranova V Mathur S Fadeel B Star A Kagan VE Shvedova AA Graphene Oxide, But Not Fullerenes, Targets Immunoproteasomes and Suppresses Antigen Presentation by Dendritic Cells Small 2012 9 1686 1690 22887961 10.1002/smll.201201546 PMC4009732

[B37] Fadel TR Li N Shah S Look M Pfefferle LD Haller GL Justesen S Wilson CJ Fahmy TM Adsorption of multimeric T cell antigens on carbon nanotubes: effect on protein structure and antigen-specific T cell stimulation Small 2013 9 666 672 10.1002/smll.201201684 23090793

[B38] Zhi X Fang H Bao C Shen G Zhang J Wang K Guo S Wan T Cui D The immunotoxicity of graphene oxides and the effect of PVP-coating Biomaterials 2013 34 5254 5261 10.1016/j.biomaterials.2013.03.024 23566800

[B39] Pescatori M Bedognetti D Venturelli E Ménard-Moyon C Bernardini C Muresu E Piana A Maida G Manetti R Sgarrella F Bianco A Delogu LG Functionalized carbon nanotubes as immunomodulator systems Biomaterials 2013 34 4395 4403 10.1016/j.biomaterials.2013.02.052 23507086

[B40] Fiorillo E Orrú V Stanford SM Liu Y Salek M Rapini N Schenone AD Saccucci P Delogu LG Angelini F Manca Bitti ML Schmedt C Chan AC Acuto O Bottini N Autoimmune-associated PTPN22 R620W variation reduces phosphorylation of lymphoid phosphatase on an inhibitory tyrosine residue J Biol Chem 2010 285 26506 26518 10.1074/jbc.M110.111104 20538612 PMC2924087

[B41] Dale DC Boxer L Liles WC The phagocytes: neutrophils and monocytes Blood 2008 112 935 945 10.1182/blood-2007-12-077917 18684880

[B42] Parodi A Quattrocchi N van de Ven AL Chiappini C Evangelopoulos M Martinez JO Brown BS Khaled SZ Yazdi IK Enzo MV Isenhart L Ferrari M Tasciotti E Synthetic nanoparticles functionalized with biomimetic leukocyte membranes possess cell-like functions Nat Nanotechnol 2013 8 61 68 23241654 10.1038/nnano.2012.212 PMC3751189

[B43] Meunier E Coste A Olagnier D Authier H Lefèvre L Dardenne C Bernad J Béraud M Flahaut E Pipy B Double-walled carbon nanotubes trigger IL-1beta release in human monocytes through Nlrp3 inflammasome activation Nanomedicine 2012 8 987 995 10.1016/j.nano.2011.11.004 22100755

[B44] Zhao D Alizadeh D Zhang L Liu W Farrukh O Manuel E Diamond DJ Badie B Carbon nanotubes enhance CpG uptake and potentiate antiglioma immunity Clin Cancer Res 2011 17 771 782 10.1158/1078-0432.CCR-10-2444 21088258 PMC3041854

[B45] Wang X Xia T Ntim SA Ji Z Lin S Meng H Chung CH George S Zhang H Wang M Li N Yang Y Castranova V Mitra S Bonner JC Nel AE Dispersal state of multiwalled carbon nanotubes elicits profibrogenic cellular responses that correlate with fibrogenesis biomarkers and fibrosis in the murine lung ACS Nano 2011 5 9772 9787 10.1021/nn2033055 22047207 PMC4136431

[B46] Gul-Uludag H Lu W Xu P Xing J Chen J Efficient and rapid uptake of magnetic carbon nanotubes into human monocytic cells: implications for cell-based cancer gene therapy Biotechnol Lett 2012 34 989 993 10.1007/s10529-012-0858-y 22286181

[B47] Li R Wang X Ji Z Sun B Zhang H Chang CH Lin S Meng H Liao YP Wang M Li Z Hwang AA Song TB Xu R Yang Y Zink JI Nel AE Xia T Surface charge and cellular processing of covalently functionalized multiwall carbon nanotubes determine pulmonary toxicity ACS Nano 2013 7 2352 2368 10.1021/nn305567s 23414138 PMC4012619

[B48] Chou CC Hsiao HY Hong QS Chen CH Peng YW Chen HW Yang PC Single-walled carbon nanotubes can induce pulmonary injury in mouse model Nano Lett 2008 8 437 445 10.1021/nl0723634 18225938

[B49] Galon J Angell HK Bedognetti D Marincola FM The continuum of cancer immunosurveillance: prognostic, predictive, and mechanistic signatures Immunity 2013 39 11 26 10.1016/j.immuni.2013.07.008 23890060

[B50] Bedognetti D Spivey TL Zhao Y Uccellini L Tomei S Dudley ME Ascierto ML De Giorgi V Liu Q Delogu LG Sommariva M Sertoli MR Simon R Wang E Rosenberg SA Marincola FM CXCR3/CCR5 pathways in metastatic melanoma patients treated with adoptive therapy and interleukin-2 Br J Cancer 2013 109 2412 2423 10.1038/bjc.2013.557 24129241 PMC3817317

[B51] Spivey TL Uccellini L Ascierto ML Zoppoli G De Giorgi V Delogu LG Engle AM Thomas JM Wang E Marincola FM Bedognetti D Gene expression profiling in acute allograft rejection: challenging the immunologic constant of rejection hypothesis J Transl Med 2011 9 174 10.1186/1479-5876-9-174 21992116 PMC3213224

[B52] Ascierto ML De Giorgi V Liu Q Bedognetti D Spivey TL Murtas D Uccellini L Ayotte BD Stroncek DF Chouchane L Manjili MH Wang E Marincola FM An immunologic portrait of cancer J Transl Med 2011 9 146 10.1186/1479-5876-9-146 21875439 PMC3175185

[B53] Yang R Yang X Zhang Z Zhang Y Wang S Cai Z Jia Y Ma Y Zheng C Lu Y Roden R Chen Y Single-walled carbon nanotubes-mediated in vivo and in vitro delivery of siRNA into antigen-presenting cells Gene Ther 2006 13 1714 1723 10.1038/sj.gt.3302808 16838032

[B54] Dutta D Sundaram SK Teeguarden JG Riley BJ Fifield LS Jacobs JM Addleman SR Kaysen GA Moudgil BM Weber TJ Adsorbed proteins influence the biological activity and molecular targeting of nanomaterials Toxicol Sci 2007 100 303 315 10.1093/toxsci/kfm217 17709331

[B55] Porter AE Gass M Muller K Skepper JN Midgley PA Welland M Direct imaging of single-walled carbon nanotubes in cells Nat Nanotechnol 2007 2 713 717 10.1038/nnano.2007.347 18654411

[B56] Schipper ML Nakayama-Ratchford N Davis CR Kam NW Chu P Liu Z Sun X Dai H Gambhir SS A pilot toxicology study of single-walled carbon nanotubes in a small sample of mice Nat Nanotechnol 2008 3 216 221 10.1038/nnano.2008.68 18654506

[B57] VanHandel M Alizadeh D Zhang L Kateb B Bronikowski M Manohara H Badie B Selective uptake of multi-walled carbon nanotubes by tumor macrophages in a murine glioma model J Neuroimmunol 2009 208 3 9 10.1016/j.jneuroim.2008.12.006 19181390

[B58] Porter AE Gass M Bendall JS Muller K Goode A Skepper JN Midgley PA Welland M Uptake of noncytotoxic acid-treated single-walled carbon nanotubes into the cytoplasm of human macrophage cells ACS Nano 2009 3 1485 1492 10.1021/nn900416z 19459622

[B59] Konduru NV Tyurina YY Feng W Basova LV Belikova NA Bayir H Clark K Rubin M Stolz D Vallhov H Scheynius A Witasp E Fadeel B Kichambare PD Star A Kisin ER Murray AR Shvedova AA Kagan VE Phosphatidylserine targets single-walled carbon nanotubes to professional phagocytes in vitro and in vivo PLoS One 2009 4 e4398 10.1371/journal.pone.0004398 19198650 PMC2634966

[B60] Antonelli A Serafini S Menotta M Sfara C Pierigé F Giorgi L Ambrosi G Rossi L Magnani M Improved cellular uptake of functionalized single-walled carbon nanotubes Nanotechnology 2010 21 425101 10.1088/0957-4484/21/42/425101 20858931

[B61] Crinelli R Carloni E Menotta M Giacomini E Bianchi M Ambrosi G Giorgi L Magnani M Oxidized ultrashort nanotubes as carbon scaffolds for the construction of cell-penetrating NF-kappaB decoy molecules ACS Nano 2010 4 2791 2803 10.1021/nn100057c 20411956

[B62] Klaper R Arndt D Setyowati K Chen J Goetz F Functionalization impacts the effects of carbon nanotubes on the immune system of rainbow trout Oncorhynchus mykiss Aquat Toxicol 2010 100 211 217 10.1016/j.aquatox.2010.07.023 20732719

[B63] Zhou F Xing D Wu B Wu S Ou Z Chen WR New insights of transmembranal mechanism and subcellular localization of noncovalently modified single-walled carbon nanotubes Nano Lett 2010 10 1677 1681 10.1021/nl100004m 20369892 PMC6005367

[B64] Meng J Yang M Jia F Xu Z Kong H Xu H Immune responses of BALB/c mice to subcutaneously injected multi-walled carbon nanotubes Nanotoxicology 2011 5 583 591 10.3109/17435390.2010.523483 21034373

[B65] Deng X Xiong D Wang Y Chen W Luan Q Zhang H Jiao Z Wu M Water soluble multi-walled carbon nanotubes enhance peritoneal macrophage activity in vivo J Nanosci Nanotechnol 2010 10 8663 8669 10.1166/jnn.2010.2684 21121380

[B66] Kagan VE Konduru NV Feng W Allen BL Conroy J Volkov Y Vlasova II Belikova NA Yanamala N Kapralov A Tyurina YY Shi J Kisin ER Murray AR Franks J Stolz D Gou P Klein-Seetharaman J Fadeel B Star A Shvedova AA Carbon nanotubes degraded by neutrophil myeloperoxidase induce less pulmonary inflammation Nat Nanotechnol 2010 5 354 359 10.1038/nnano.2010.44 20364135 PMC6714564

[B67] Montes-Fonseca SL Orrantia-Borunda E Aguilar-Elguezabal A González Horta C Talamás-Rohana P Sánchez-Ramírez B Cytotoxicity of functionalized carbon nanotubes in J774A macrophages Nanomedicine 2012 8 853 859 10.1016/j.nano.2011.10.002 22033080

[B68] Gao N Zhang Q Mu Q Bai Y Li L Zhou H Butch ER Powell TB Snyder SE Jiang G Yan B Steering carbon nanotubes to scavenger receptor recognition by nanotube surface chemistry modification partially alleviates NFkappaB activation and reduces its immunotoxicity ACS Nano 2011 5 4581 4591 10.1021/nn200283g 21595480 PMC3138538

[B69] Roda E Coccini T Acerbi D Barni S Vaccarone R Manzo L Comparative pulmonary toxicity assessment of pristine and functionalized multi-walled carbon nanotubes intratracheally instilled in rats: morphohistochemical evaluations Histol Histopathol 2011 26 357 367 21210349 10.14670/HH-26.357

[B70] Tahara Y Miyawaki J Zhang M Yang M Waga I Iijima S Irie H Yudasaka M Histological assessments for toxicity and functionalization-dependent biodistribution of carbon nanohorns Nanotechnology 2011 22 265106 10.1088/0957-4484/22/26/265106 21586808

[B71] Prajapati VK Awasthi K Gautam S Yadav TP Rai M Srivastava ON Sundar S Targeted killing of Leishmania donovani in vivo and in vitro with amphotericin B attached to functionalized carbon nanotubes J Antimicrob Chemother 2011 66 874 879 10.1093/jac/dkr002 21393222 PMC3307040

[B72] Tabet L Bussy C Setyan A Simon-Deckers A Rossi MJ Boczkowski J Lanone S Coating carbon nanotubes with a polystyrene-based polymer protects against pulmonary toxicity Part Fibre Toxicol 2011 8 3 10.1186/1743-8977-8-3 21255417 PMC3030506

[B73] Al-Jamal KT Nerl H Müller KH Ali-Boucetta H Li S Haynes PD Jinschek JR Prato M Bianco A Kostarelos K Porter AE Cellular uptake mechanisms of functionalised multi-walled carbon nanotubes by 3D electron tomography imaging Nanoscale 2011 3 2627 2635 10.1039/c1nr10080g 21603701

[B74] Boncel S Muller KH Skepper JN Walczak KZ Koziol KK Tunable chemistry and morphology of multi-wall carbon nanotubes as a route to non-toxic, theranostic systems Biomaterials 2011 32 7677 7686 10.1016/j.biomaterials.2011.06.055 21764122

[B75] Patlolla AK Berry A Tchounwou PB Study of hepatotoxicity and oxidative stress in male Swiss-Webster mice exposed to functionalized multi-walled carbon nanotubes Mol Cell Biochem 2011 358 189 199 10.1007/s11010-011-0934-y 21725842 PMC3768273

[B76] Kosuge H Sherlock SP Kitagawa T Terashima M Barral JK Nishimura DG Dai H McConnell MV FeCo/graphite nanocrystals for multi-modality imaging of experimental vascular inflammation PLoS One 2011 6 e14523 10.1371/journal.pone.0014523 21264237 PMC3021517

[B77] Dong PX Wan B Guo LH In vitro toxicity of acid-functionalized single-walled carbon nanotubes: effects on murine macrophages and gene expression profiling Nanotoxicology 2012 6 288 303 10.3109/17435390.2011.573101 21486190

[B78] Zhang M Zhou X Iijima S Yudasaka M Small-sized carbon nanohorns enabling cellular uptake control Small 2012 8 2524 2531 10.1002/smll.201102595 22674899

[B79] Tahara Y Nakamura M Yang M Zhang M Iijima S Yudaska M Lysosomal membrane destabilization induced by high accumulation of single-walled carbon nanohorns in murine macrophage RAW 264.7 Biomaterials 2012 33 2762 2769 10.1016/j.biomaterials.2011.12.023 22209643

[B80] Chen T Zang J Wang H Nie H Wang X Shen Z Tang S Yang J Jia G Water-soluble taurine-functionalized multi-walled carbon nanotubes induce less damage to mitochondria of RAW 264.7 cells J Nanosci Nanotechnol 2012 12 8008 8016 10.1166/jnn.2012.6593 23421171

[B81] Zhang T Tang M Kong L Li H Zhang T Zhang S Xue Y Pu Y Comparison of cytotoxic and inflammatory responses of pristine and functionalized multi-walled carbon nanotubes in RAW 264.7 mouse macrophages J Hazard Mater 2012 219–220 203 212 10.1016/j.jhazmat.2012.03.079 22534157

[B82] Luo M Deng X Shen X Dong L Liu Y Comparison of cytotoxicity of pristine and covalently functionalized multi-walled carbon nanotubes in RAW 264.7 macrophages J Nanosci Nanotechnol 2012 12 274 283 10.1166/jnn.2012.5700 22523976

[B83] Clark KA O'Driscoll C Cooke CA Smith BA Wepasnick K Fairbrother DH Lees PS Bressler JP Evaluation of the interactions between multiwalled carbon nanotubes and Caco-2 cells J Toxicol Environ Health A 2012 75 25 35 10.1080/15287394.2011.589105 22047161

[B84] Schinwald A Murphy FA Jones A MacNee W Donaldson K Graphene-based nanoplatelets: a new risk to the respiratory system as a consequence of their unusual aerodynamic properties ACS Nano 2012 6 736 746 10.1021/nn204229f 22195731

[B85] Li Y Liu Y Fu Y Wei T Le Guyader L Gao G Liu RS Chang YZ Chen C The triggering of apoptosis in macrophages by pristine graphene through the MAPK and TGF-beta signaling pathways Biomaterials 2012 33 402 411 10.1016/j.biomaterials.2011.09.091 22019121

[B86] Chen GY Yang HJ Lu CH Chao YC Hwang SM Chen CL Lo KW Sung LY Luo WY Tuan HY Hu YC Simultaneous induction of autophagy and toll-like receptor signaling pathways by graphene oxide Biomaterials 2012 33 6559 6569 10.1016/j.biomaterials.2012.05.064 22704844

[B87] Yue H Wei W Yue Z Wang B Luo N Gao Y Ma D Ma G Su Z The role of the lateral dimension of graphene oxide in the regulation of cellular responses Biomaterials 2012 33 4013 4021 10.1016/j.biomaterials.2012.02.021 22381473

[B88] Zhou H Zhao K Li W Yang N Liu Y Chen C Wei T The interactions between pristine graphene and macrophages and the production of cytokines/chemokines via TLR- and NF-kappaB-related signaling pathways Biomaterials 2012 33 6933 6942 10.1016/j.biomaterials.2012.06.064 22796167

[B89] Rawson FJ Yeung CL Jackson SK Mendes PM Tailoring 3D single-walled carbon nanotubes anchored to indium tin oxide for natural cellular uptake and intracellular sensing Nano Lett 2013 13 1 8 10.1021/nl203780d 22268573 PMC3542912

[B90] Matesanz MC Vila M Feito MJ Linares J Gonçalves G Vallet-Regi M Marques PA Portolés MT The effects of graphene oxide nanosheets localized on F-actin filaments on cell-cycle alterations Biomaterials 2013 34 1562 1569 10.1016/j.biomaterials.2012.11.001 23177613

[B91] Yang M Wada M Zhang M Kostarelos K Yuge R Iijima S Masuda M Yudasaka M A high poly(ethylene glycol) density on graphene nanomaterials reduces the detachment of lipid-poly(ethylene glycol) and macrophage uptake Acta Biomater 2013 9 4744 4753 10.1016/j.actbio.2012.09.012 22995407

[B92] Hamilton RF Jr Xiang C Li M Ka I Yang F Ma D Porter DW Wu N Holian A Purification and sidewall functionalization of multiwalled carbon nanotubes and resulting bioactivity in two macrophage models Inhal Toxicol 2013 25 199 210 10.3109/08958378.2013.775197 23480196 PMC4127292

[B93] Girish CM Sasidharan A Gowd GS Nair S Koyakutty M Confocal Raman Imaging Study Showing Macrophage Mediated Biodegradation of Graphene In Vivo Adv Healthc Mater 2013 2 1489 1500 10.1002/adhm.201200489 23554400

[B94] Dvash R Khatchatouriants A Solmesky LJ Wibroe PP Weil M Moghimi SM Peer D Structural profiling and biological performance of phospholipid-hyaluronan functionalized single-walled carbon nanotubes J Control Release 2013 170 295 305 10.1016/j.jconrel.2013.05.042 23764531

[B95] Wan B Wang ZX Lv QY Dong PX Zhao LX Yang Y Guo LH Single-walled carbon nanotubes and graphene oxides induce autophagosome accumulation and lysosome impairment in primarily cultured murine peritoneal macrophages Toxicol Lett 2013 221 118 127 10.1016/j.toxlet.2013.06.208 23769962

[B96] Jiang Y Zhang H Wang Y Chen M Ye S Hou Z Ren L Modulation of apoptotic pathways of macrophages by surface-functionalized multi-walled carbon nanotubes PLoS One 2013 8 e65756 10.1371/journal.pone.0065756 23755279 PMC3675050

[B97] Qu G Liu S Zhang S Wang L Wang X Sun B Yin N Gao X Xia T Chen JJ Jiang GB Graphene oxide induces toll-like receptor 4 (TLR4)-dependent necrosis in macrophages ACS Nano 2013 7 5732 5745 10.1021/nn402330b 23734789

[B98] Villegas JC Alvarez-Montes L Rodríguez-Fernández L González J Valiente R Fanarraga ML Multiwalled Carbon Nanotubes Hinder Microglia Function Interfering with Cell Migration and Phagocytosis Adv Healthc Mater 2013 3 424 432 23950018 10.1002/adhm.201300178

[B99] Li Y Yuan H von dem Bussche A Creighton M Hurt RH Kane AB Gao H Graphene microsheets enter cells through spontaneous membrane penetration at edge asperities and corner sites Proc Natl Acad Sci U S A 2013 110 12295 12300 10.1073/pnas.1222276110 23840061 PMC3725082

[B100] Kotagiri N Lee JS Kim JW Selective pathogen targeting and macrophage evading carbon nanotubes through dextran sulfate coating and PEGylation for photothermal theranostics J Biomed Nanotechnol 2013 9 1008 1016 10.1166/jbn.2013.1531 23858965

[B101] Russier J Treossi E Scarsi A Perrozzi F Dumortier H Ottaviano L Meneghetti M Palermo V Bianco A Evidencing the mask effect of graphene oxide: a comparative study on primary human and murine phagocytic cells Nanoscale 2013 5 11234 11247 10.1039/c3nr03543c 24084792

[B102] Yang M Flavin K Kopf I Radics G Hearnden CH McManus GJ Moran B Villalta-Cerdas A Echegoyen LA Giordani S Lavelle EC Functionalization of Carbon Nanoparticles Modulates Inflammatory Cell Recruitment and NLRP3 Inflammasome Activation Small 2013 9 4194 4206 10.1002/smll.201300481 23839951

[B103] Lacotte S García A Décossas M Al-Jamal WT Li S Kostarelos K Muller S Prato M Dumortier H Alberto B Interfacing Functionalized Carbon Nanohorns with Primary Phagocytic Cells Adv Materials 2008 20 2421 2426 10.1002/adma.200702753

[B104] Pantarotto D Briand JP Prato M Bianco A Translocation of bioactive peptides across cell membranes by carbon nanotubes Chem Commun (Camb) 2004 16 17 10.1039/b311254c 14737310

[B105] Poland CA Duffin R Kinloch I Maynard A Wallace WA Seaton A Stone V Brown S Macnee W Donaldson K Carbon nanotubes introduced into the abdominal cavity of mice show asbestos-like pathogenicity in a pilot study Nat Nanotechnol 2008 3 423 428 10.1038/nnano.2008.111 18654567

[B106] Salvador-Morales C Flahaut E Sim E Sloan J Green ML Sim RB Complement activation and protein adsorption by carbon nanotubes Mol Immunol 2006 43 193 201 10.1016/j.molimm.2005.02.006 16199256

[B107] Ali-Boucetta HN Sainz R Herrero MA Tian B Prato M Bianco A Kostarelos K Asbestos-like pathogenicity of long carbon nanotubes alleviated by chemical functionalization Angew Chem Int Ed 2013 52 2274 2278 10.1002/anie.201207664 23319294

[B108] Coccini T Manzo L Roda E Safety evaluation of engineered nanomaterials for health risk assessment: an experimental tiered testing approach using pristine and functionalized carbon nanotubes ISRN Toxicol 2013 2013 825427 23724301 10.1155/2013/825427 PMC3658371

[B109] Beg S Rizwan M Sheikh AM Hasnain MS Anwer K Kohli K Advancement in carbon nanotubes: basics, biomedical applications and toxicity J Pharm Pharmacol 2011 63 141 163 10.1111/j.2042-7158.2010.01167.x 21235578

[B110] Bianco A Graphene: safe or toxic? The two faces of the medal Angew Chem Int Ed Engl 2013 52 4986 4997 10.1002/anie.201209099 23580235

[B111] Gottardi R Douradinha B Carbon nanotubes as a novel tool for vaccination against infectious diseases and cancer J Nanobiotechnology 2013 11 30 10.1186/1477-3155-11-30 24025216 PMC3846653

[B112] Tomei S Wang E Delogu LG Marincola FM Bedognetti D Non-BRAF-targeted therapy, immunotherapy, and combination therapy for melanoma Expert Opin Biol Ther 2014 14 663 686 10.1517/14712598.2014.890586 24625306

[B113] Bedognetti D Balwit JM Wang E Disis ML Britten CM Delogu LG Tomei S Fox BA Gajewski TF Marincola FM Butterfield LH SITC/iSBTc Cancer Immunotherapy Biomarkers Resource Document: online resources and useful tools - a compass in the land of biomarker discovery J Transl Med 2011 9 155 10.1186/1479-5876-9-155 21929757 PMC3189883

[B114] Shay T Jojic V Zuk O Rothamel K Puyraimond-Zemmour D Feng T Wakamatsu E Benoist C Koller D Regev A Conservation and divergence in the transcriptional programs of the human and mouse immune systems Proc Natl Acad Sci U S A 2013 110 2946 2951 10.1073/pnas.1222738110 23382184 PMC3581886

