# Supplementary material for: Impact of carbon nanotubes and graphene on immune cells
Source: J Transl Med. 2014 May 21;12:138. doi: 10.1186/1479-5876-12-138 (PMC4067374; doi:10.1186/1479-5876-12-138)
Supplement: Additional file 1 — Functionalized carbon nanotubes and graphene on Lymphocytes. [file 1479-5876-12-138-S1.pdf]

Table 1. Functionalized carbon nanotubes and graphene on Lymphocytes

Material	Funzionalizations	Species	Model	Lymphocyte type and other cells	Year	Reference
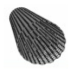	Oxidized and conjugate with FITC and amphotericin B	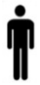	In vitro (Jurkat)	L. T	2005	Wu W et al. (Angew. Chem.)
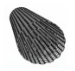	Oxidized	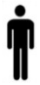	Ex vivo, in vitro (Jurkat)	L. T	2006	Bottini M et al. (Toxicology Letters)
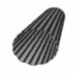	*1,3-dipolar cycloaddition and oxidation/amidation*	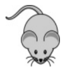	*Ex vivo*	*L. B and T, Macrophages and Polynuclear cells*	*2006*	*Dumortier H et al. (Nano Letters)*
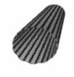	Streptavidin-conjugated Quantum Dots	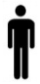	In vitro (Jurkat)	L.T	2006	Bottini M et al. (Biomacromolecules)
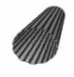	*PL-PEG and conjugated with RNAi of CXCR4 and CD4 receptors*	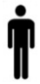	*Ex vivo, in vitro (MAGI)*	*L. T, PBMCs*	*2007*	*Liu Z et al. (Angew. Chem)*
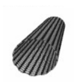	PEGylated and fluorochrome-coupled neutravidin, and subsequently with anti-CD3epsilon and anti-CD28	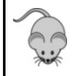 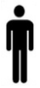	In vivo, in vitro (Jurkat)	L. T	2008	Cato MH et al. (J Nanosci Nanotechnol.)
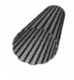	Acid treated and conjugate with antibody (anti-CD3)	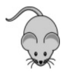	In vitro [Hybridoma T cells (B3Z)]	L. T	2008	Fadel TR et al. (Nano Letters)
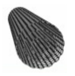	Nylon-6	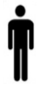	Ex vivo	PBMCs	2009	Ochoa-Olmos et al. (J Nanosci Nanotechnol)

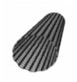	PEGylated conjugated with anti-PTPN22 20-mer antisense oligonucleotides	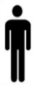	In vitro (Jurkat)	L. T	2009	Delogu LG et al. (Bioconjugate Chem.)
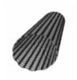	*Carboxylated and conjugated with tuberculin purified protein derivative (PDD)*	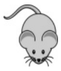	*Ex vivo, in vivo*	*L. T, Macrophages*	*2009*	*Zeinali M et al. (Immunology Letters)*
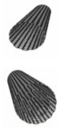	Amidated	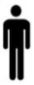	Ex vivo	PBMCs	2010	Cveticanin J et al. (Nanotechnology)
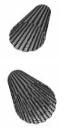	Treated with Tween 80	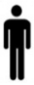	In vitro (A3 L.)	L. T	2010	Hu X et al. (Science of the Total Environment)
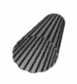	Acid treated and conjugate with antibody (CD3 and CD28)	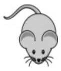	Ex vivo	L. T	2010	Fadel TR et al. (Langmuir)
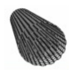	Oxidized	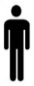	In vitro (Jurkat)	L. T	2010	Sabuncu AC et al. (Colloids and Surfaces B: Biointerfaces)
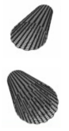	PEGylated/TEGylated and conjugate with Amphotericin B	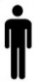	In vitro (Jurkat)	L. T	2010	Benincasa M et al. (ACS Nano)
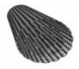	*Acid treated*	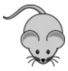	*In vivo*	*L. T, Macrophages*	*2010*	*Kim JE et al. (J Toxicol Environ Health)*
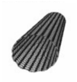	PEGylated	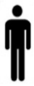	In vitro (Jurkat)	L. T	2010	Delogu LG et al. (J Nanosci Nanotechnol)

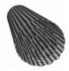	Oxidized	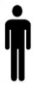	Ex vivo	PBMCs	2011	Sun Z et al. (Plos One)
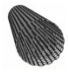	*Oxidized, amidated and conjuged with FITC*	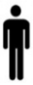	*Ex vivo*	*PBMCs*	*2012*	*Delogu LG et al. (Nanomedicine (Lond).)*
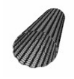	*Carboxylated*	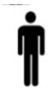	*Ex vivo, in vivo*	*L. T, DCs*	*2011*	*Tkach AV et al. (ACS Nano)*
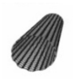	*Primary amines conjugate with WT1Pep427*	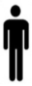	*Ex vivo*	*L. T, DCs*	*2011*	*Villa CH (ACS Nano)*
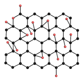	None	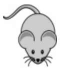	In vivo, ex vivo	L. T	2012	Ni G et al. (Immunology letters)
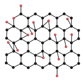	*None*	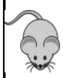 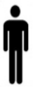	*Ex vivo, in vitro (RAW 264.7)*	*PBMCs, Macrophages*	*2012*	*Sasidharan A et al. (Small)*
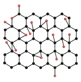	*Negatively charged*	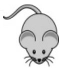	*Ex vivo, in vitro [Hybridoma T cells (B3Z)]*	*L. T, DCs*	*2012*	*Tkach AV et al. (Small)*
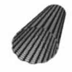	Carboxylated and conjugate with MHC-I/SIIN	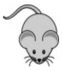	Ex vivo	L. T	2013	Fadel TR (Small)
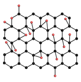 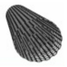	*Oxidized and 1,3-dipolar cycloaddition*	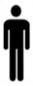	*In vitro (Jurkat, THP1)*	*L. T, Monocytes*	*2013*	*Pescatori M et al. (Biomaterials)*

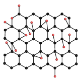	*Coated with polyvinylpyrrolidone (PVP)*	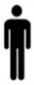	*Ex vivo*	*L. T, DCs, Macrophages*	*2013*	*Zhi X et al. (Biomaterials)*

Legend	
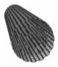	MWCNTs
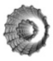	DWCNTs
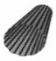	SWCNTs
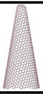	Carbon Nanohorns
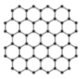	Graphene
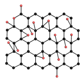	Graphene Oxide
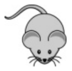	Mouse
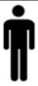	Human
Italics	*Articles that considered more than one type of cells*
