# Supplementary material for: Impact of carbon nanotubes and graphene on immune cells
Source: J Transl Med. 2014 May 21;12:138. doi: 10.1186/1479-5876-12-138 (PMC4067374; doi:10.1186/1479-5876-12-138)
Supplement: Additional file 2 — Functionalized carbon nanotubes and graphene on Monocytes. [file 1479-5876-12-138-S2.pdf]

Table 2. Functionalized carbon nanotubes and graphene on Monocytes

Material	Funzionalizations	Species	Model	Other cell types	Year	Reference
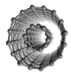	Oxidized	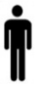	Ex vivo		2012	Meunier E et al. (Nanomedicine)
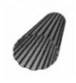	PEGylated and coniugate with CpG	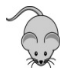	Ex vivo		2011	Zhao D et al. (Clin Cancer Res.)
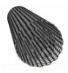	*Oxidized, amidized conjuged with FITC*	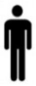	*Ex vivo*	*PBMCs*	*2012*	*Delogu LG et al. (Nanomedicine (Lond).)*
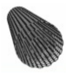	*Carboxylated*	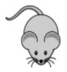	*In vivo, in vitro (THP1)*	*Macrophages*	*2011*	*Wang X et al. (ACS Nano)*
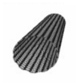	FITC	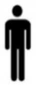	In vitro (THP1)		2012	Gul-Uludag H et al. (Biotecnol. Let.)
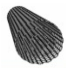	*Oxidized and functionalized with ammonium group and FITC*	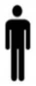	*In vitro (THP1)*	*L. T*	*2013*	*Pescatori M et al. (Biomaterials)*
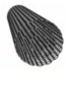	*Carboxylated, PEGylated, aminated, sidewall aminated, and PEI-modified*	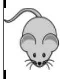 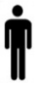	*In vivo, in vitro (THP1)*	*Macrophages*	*2013*	*Li R et al. (ACS Nano)*

Legend	
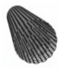	MWCNTs
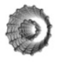	DWCNTs
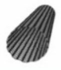	SWCNTs
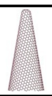	Carbon Nanohorns
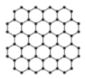	Graphene
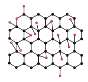	Graphene Oxide
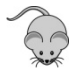	Mouse
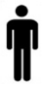	Human
Italics	*Articles that considered more than one type of cells*
