# Supplementary material for: Impact of carbon nanotubes and graphene on immune cells
Source: J Transl Med. 2014 May 21;12:138. doi: 10.1186/1479-5876-12-138 (PMC4067374; doi:10.1186/1479-5876-12-138)
Supplement: Additional file 3 — Functionalized carbon nanotubes and graphene on Macrophages. [file 1479-5876-12-138-S3.pdf]

Table 3. Functionalized carbon nanotubes and graphene on Macrophages

Material	Funzionalizations	Species	Model	Other cell types	Year	Reference
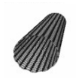	*DNA and siRNA functionalizated*	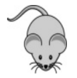	*In vivo In vitro (RAW 264.7)*	*DCs*	*2006*	*Yang R et al. (Gene Therapy)*
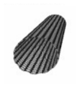	*1,3-dipolar cycloaddition and oxidation/amidation*	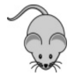	*Ex vivo*	*L. B and T, Macrophages and Polynuclear cells*	*2006*	*Dumortier H et al. (Nano Letters)*
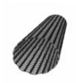	Oxidized	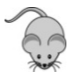	In vitro (RAW 264.7)		2007	Dutta D et al. (Toxicological Science)
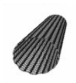	Silver iodide	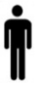	Ex vivo		2007	Porter AE et al. (Nat Nanotechnol.)
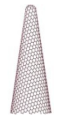	Amination and FITC conjugation	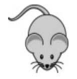	Ex vivo		2008	Lacotte S. et al. (Adv. Materials)
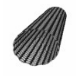	PEGylated	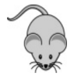	in vivo		2008	Schipper ML (Nat Nanotechnol.)
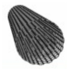	PKH 26	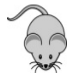	In vivo		2009	VanHandel M et al. (Neuroimmunology)
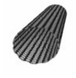	Acid-treated	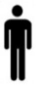	Ex vivo		2009	Porter AE et al. (ACS Nano)

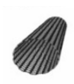	*Phospholipids PC [pro-apoptotic cargo (cytochrome c)]*	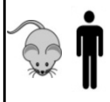	*In vivo, ex vivo, in vitro (RAW 264.7)*	*DCs, Brain microglia*	*2009*	*Konduru NV et al. (Plos One)*
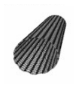	*Tuberculin purified protein derivative (PPD)*	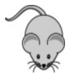	*Ex vivo, in vivo*	*L. T, Macrophages*	*2009*	*Zeinali M et al. (Immunology Letters)*
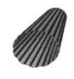	Oxidized and coated with stearyl alcohol, and derivates coated with phosphatidylethanolamine (PE) or phosphatidylserine (PS)	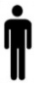	Ex vivo, in vitro (U937 monoblastoid)		2010	Antonelli A et al. (Nanotechnology.)
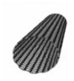	Oligonucleotide against NF-kB	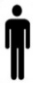	Ex vivo		2010	Crinelli R et al. (ACS Nano)
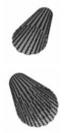	Polyaminobenzene sulfonic acids (PABS), sulfonate groups/PEG groups	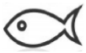	Ex vivo		2010	Klaper R et al. (Aquatic Toxicology)
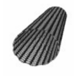	PL-PEGylated	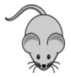	In vitro (RAW 264.7)		2010	Zhou F et al. (NANO Letters)
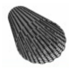	Oxidized	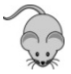	In vivo		2010	Meng J et al. (Nanotoxicology)
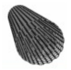	Oxidized	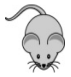	In vivo		2010	Deng X et al. (J Nanosci Nanotechnol.)
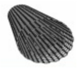	*Acid treated*	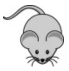	*In vivo*	*L. T*	*2010*	*Kim JE et al. (J Toxicol Environ Health)*
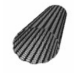	Carboxylated	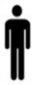	Ex vivo		2010	Kagan VE et al. (Nat Nanotechnol.)

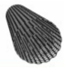	*Carboxylated*	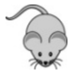	*In vivo, in vitro (THP1)*	*Monocytes*	*2011*	*Wang X et al. (ACS Nano)*
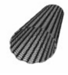	N-Boc-2,2-dioxyethylene diethylamine linker and FITC/L220	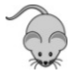	In vitro (J774A)		2011	Montes-Fonseca SL et al. (Nanomedicine)
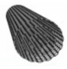	Carboxylated	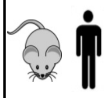	In vivo, in vitro (THP1)		2011	Gao N et al. (ACS Nano)
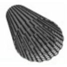	Carboxylated/ammine-containing nanotubes	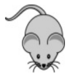	In vivo		2011	Roda E et al. (Histol Histopathol.)
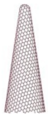	Oxidized/BSA	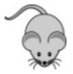	In vivo		2011	Tahara Y et al. (Nanotechnology)
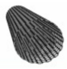	Amphotericin B	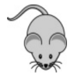	In vivo, in vitro (J774A.1)		2011	Prajapati VK et al. (J Antimicrob Chemother.)
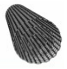	Acid- and polystyrene-based	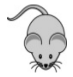	In vivo, in vitro (RAW 264.7)		2011	Tabet L et al. (Particle and Fibre Toxicology)
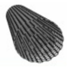	Ammonium functionalized	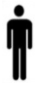	Ex vivo		2011	Al-Jamal KT et al. (Nanoscale)
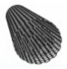	Oxidized and N-doped	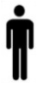	Ex vivo		2011	Boncel S et al. (Biomaterials)
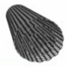	Carboxylated	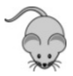	In vivo		2011	Patlolla AK et al. (Mol Cell Biochem)

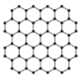	FeCo nanocrystal	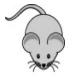	In vivo		2011	Kosuge H et al. (Plos One)
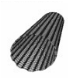	Acid-treated	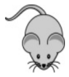	In vitro (RAW 264.7)		2012	Dong PX et al. (Nanotoxicology)
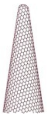	PEGylated/Tumor targetin folic acid (FA)	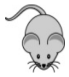	In vitro (RAW 264.7)		2012	Zhang M et al (Small)
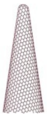	Oxidized	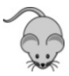	In vitro (RAW 264.7)		2012	Tahara Y et al. (Biomaterials)
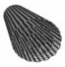	Taurine	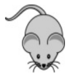	In vitro (RAW 264.7)		2012	Chen T et al. (J Nanosci Nanotechnol)
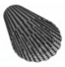	Carboxylated/PEGylated	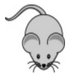	In vitro (RAW 264.7)		2012	Zhang T et al. (J Haz Mat)
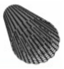	Taurine	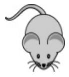	In vItro (RAW 264.7)		2012	Luo M et al. (J Nanosci Nanotechnol.)
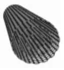	Oxidized	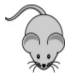	In vitro (RAW 264.7)		2012	Clark KA et al. (J Toxicol Environ Health A.)
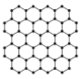	Nanoplatelets	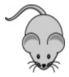	In vivo, in vitro (THP1)		2012	Schinwald A et al. (ACS Nano)
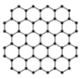	None	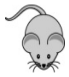	In vitro (RAW 264.7)		2012	Li Y et al. (Biomaterials)

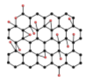	None	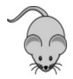	In vitro (RAW 264.7)		2012	Chen GY et al. (Biomaterials)
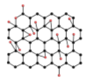	*None*	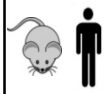	*Ex vivo, in vitro (RAW 264.7)*	*PBMCs*	*2012*	*Sasidharan A et al. (Small)*
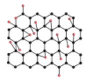	None	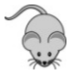	In vivo, in vitro (J774A.1)		2012	Yue H et al. (Biomaterials)
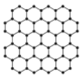	None	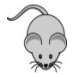	Ex vivo, in vitro (RAW 264.7)		2012	Zhou H et al (Biomaterials)
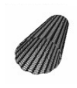	Indium tin oxide (ITO)	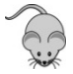	In vitro (RAW 264.7)		2013	Rawson FJ et al. (NANO Letters)
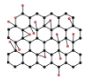	None	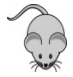	In vitro (RAW 264.7)		2013	Matesanz MC et al. (Biomaterials)
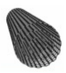	*Carboxylated, PEGylated, aminated, sidewall aminated, and PEI-modified*	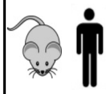	*In vivo, in vitro (THP1)*	*Monocytes*	*2013*	*Li R et al (ACS Nano)*
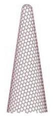	Amphiphilic lipid–poly(ethylene glycol) (LPEG)	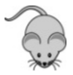	In vitro (RAW 264.7)		2013	Yang M et al. (Acta Biomaterialia)
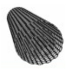	Carboxylated	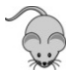	Ex vivo		2013	Hamilton RF Jr et al. (Inhal Toxicol.)
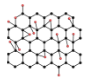	Carboxylated	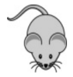	In vivo, in vitro (RAW 264.7)		2013	Girish CM et al (Adv Healthc Mater)

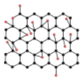	*Coated with polyvinylpyrrolidone (PVP)*	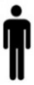	*Ex vivo*	*L. T, DCs*	*2013*	*Zhi X et al. (Biomaterials)*
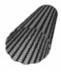	Phospholipids (PL) conjugated to hyaluronan (HA)	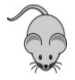	In vivo, in vitro (RAW 264.7)		2013	Dvash R et al. (Journal of Controlled Release)
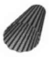 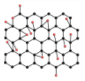	Acid funtionalized - None	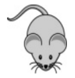	Ex vivo		2013	Wan B et al. (Toxicology Letters)
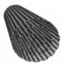	Carboxylated-PEGylated	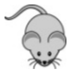	Ex vivo, in vitro (RAW 264.7)		2013	Jiang Y et al. (Plos One)
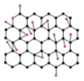	Oxidized	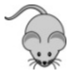	Ex vivo, in vitro (J774A 1, RAW264.7)		2013	Qu G et al. (ACS Nano)
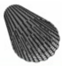	Treated with Horse Serum	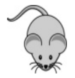	In vitro (BV2)		2013	Villegas JC et al. (Adv. Healthcare Mater.)
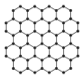	Microsheets	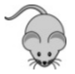	In vitro (J774)		2013	Li Y et al. (PNAS)
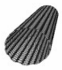	Dextran sulfate (DS)-PEGylated	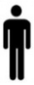	Ex vivo		2013	Kotagiri N et al. (J Biomed Nanotechnol)
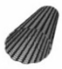	*Oxidized - benzoic acid functionalization and FITC*	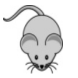	*In vivo, ex vivo*	*DCs*	*2013*	*Yang M et al. (Small)*
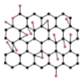	None	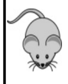 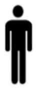	Ex vivo		2013	Russier J et al. (Nanoscale)

Legend	
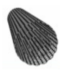	MWCNTs
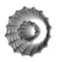	DWCNTs
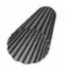	SWCNTs
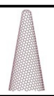	Carbon Nanohorns
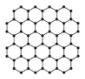	Graphene
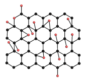	Graphene Oxide
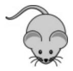	Mouse
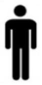	Human
Italics	*Articles that considered more than one type of cells*
