# Supplementary material for: Impact of carbon nanotubes and graphene on immune cells
Source: J Transl Med. 2014 May 21;12:138. doi: 10.1186/1479-5876-12-138 (PMC4067374; doi:10.1186/1479-5876-12-138)
Supplement: Additional file 4 — Functionalized carbon nanotubes and graphene on Dendritic cells (DCs). [file 1479-5876-12-138-S4.pdf]

Table 4. Functionalized carbon nanotubes and graphene on Dendritic cells (DCs)

Material	Funzionalizations	Species	Model	Other cell types	Year	Reference
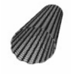	*DNA and siRNA functionalizated*	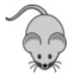	*In vivo In vitro (RAW 264.7 , Bone marrow DCs)*	*Macrophages*	*2006*	*Yang R et al. (Gene Therapy)*
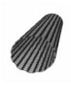	*Phospholipids [pro-apoptotic cargo (cytochrome c)]*	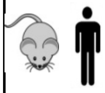	*In vivo, ex vivo, in vitro (RAW 264.7)*	*Macrophages, Brain microglia*	*2009*	*Konduru NK et al. (Plos One)*
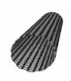	*Carboxylated*	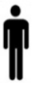	*Ex vivo, in vivo*	*L. T*	*2011*	*Tkach AV et al. (ACS Nano)*
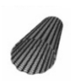	*Primary amines conjugate with WT1Pep427*	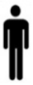	*Ex vivo*	*L. T*	*2011*	*Villa CH et al. (ACS Nano)*
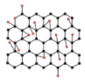	*Negatively-charged*	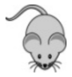	*Ex vivo, in vitro [Hybridoma T cells (B3Z)]*	*L. T*	*2012*	*Tkach AV et al. (Small)*
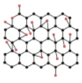	*Coated with polyvinylpyrrolidone (PVP)*	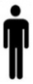	*Ex vivo*	*L. T, Macrophages*	*2013*	*Zhi X et al. (Biomaterials)*
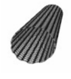	*Oxidized - benzoic acid functionalization and FITC*	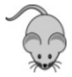	*In vivo, ex vivo*	*Macrophages*	*2013*	*Yang M et al. (Small)*

Legend	
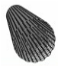	MWCNTs
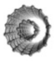	DWCNTs
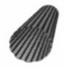	SWCNTs
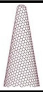	Carbon Nanohorns
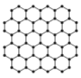	Graphene
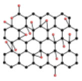	Graphene Oxide
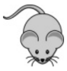	Mouse
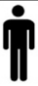	Human
Italics	*Articles that considered more than one type of cells*
